# Incidence and survival of multiply revised knee arthroplasties in Denmark 1998–2021: a nationwide register-based study

**DOI:** 10.2340/17453674.2024.41257

**Published:** 2024-08-21

**Authors:** Julius T HALD, Anders B EL-GALALY, Michael M PETERSEN, Martin LINDBERG-LARSEN, Robin CHRISTENSEN, Anders ODGAARD

**Affiliations:** 1 Department of Orthopedic Surgery, Rigshospitalet, Copenhagen University; 2 Department of Clinical Medicine, University of Copenhagen, Copenhagen; 3 Department of Orthopedic Surgery and Traumatology, Odense University Hospital; 4 Section for Biostatistics and Evidence-Based Research, the Parker Institute, Bispebjerg and Frederiksberg Hospital, Copenhagen; 5 Research Unit of Rheumatology, Department of Clinical Research, University of Southern Denmark, Odense University Hospital, Denmark

## Abstract

**Purpose:**

The primary aim of our study was to identify the absolute incidence and implant survival of multiply revised knee arthroplasties based on nationwide register data. The secondary aim was to determine the change in the absolute incidence and implant survival of multiply revised knee arthroplasties.

**Methods:**

We performed a retrospective observational study of primary knee arthroplasties using several nationwide Danish registers. All primary knee arthroplasties performed in Denmark from 1998 to 2021 were identified. From these primary arthroplasties, revision procedures were identified. Kaplan–Meier plots were used in survival analysis to estimate the likelihood of implant survival.

**Results:**

161,384 primary knee arthroplasties and their revisions performed between 1998 and 2021 were identified; of 13,786 (8.5%) revisions there were 10,638 1st revisions, 2,148 2nd revisions, 624 3rd revisions, 223 4th revisions, and 153 procedures that had been revised more than 4 times. The 10-year revision-free survival of primary arthroplasties was 92.3% (95% confidence interval [CI] 92.2–92.5). First-time revisions had a 10-year revision-free survival of 75.9% (CI 74.9–76.9). The 10-year survival of second- and third-time revisions was 65.1% (CI 62.6–67.6) and 57.8% (CI 53.4–62.5), respectively. The 10-year implant survival probabilities of primary knee arthroplasties were 91.4% in 1998–2009 and 93.3% in 2010–2021 (difference 2.2%). The 10-year implant survival probabilities of 1st revisions were 77% in 1998–2009 and 75% in 2010–2021 (difference –2.4%).

**Conclusion:**

We found that 0.3% of all primary knee arthroplasties resulted in 3 or more revisions. The implant survival decreased for each consecutive revision, with almost half of the 3rd revisions being re-revised within 10 years. The 10-survival of the primary implant was higher in 2010–2021, and the 10-year survival of the 1st revision was higher in 1998–2009.

Primary and revision knee arthroplasties are performed increasingly and more patients are expected to undergo multiple implant revisions within their lifetime [1-3]. These patients represent a complex population with severe complications, disabilities, and healthcare costs, and they pose a challenge to both surgeons and healthcare systems in general [4]. Healthcare systems will require increasing resources to address this problem, but most importantly more knowledge is needed. Studies have predicted a rise in the number of revision arthroplasties in general [2], but little is known about the historical development of re-revisions following the first revision. Neither the incidence of multiply revised total knee arthroplasties (TKA) nor how implant survival is affected by subsequent revisions have been fully investigated. Research specifically into multiply revised knee arthroplasty patients has been limited with only 1 study reporting that implant survival decreases significantly for each consecutive revision [5]. A limited number of studies have found that male sex, younger age, and certain indications for revision decrease implant survival of re-revised knee arthroplasties [5-8].

The primary aim of our study was to identify the absolute incidence and implant survival of multiply revised knee arthroplasties in Denmark from 1998 to 2021 based on nationwide register data. The secondary aim was to determine the change of the absolute incidence and implant survival of multiply revised knee arthroplasties in Denmark over the observation period.

## Methods

### Study design

This study is a retrospective observational register study and the reporting complies with the STROBE statement [9]. A detailed protocol was created and signed before data collection and analysis (available from clinicaltrials.gov, identifier: NCT06064318).

### Data sources

3 nationwide Danish registers were used: the Danish National Patient Register (DNPR), the Danish Civil Registration System (DCRS), and the Danish Knee Arthroplasty Register (DKAR). The DNPR is a national database administered by the Danish Health Authority [10]. It contains administrative information on all patient contacts from public and private institutions, including entries for all surgical procedures performed in Denmark. The DCRS is also a national administrative database collecting information regarding date of birth, sex, death, and migration [11]. The DKAR is a nationwide clinical quality database [12]. Records are submitted to the DKAR using knee-specific forms that are completed by the surgeon postoperatively. All orthopedic departments, including private institutions, are required to report data to the DKAR, ensuring high completeness of the collected data. The completeness of DKAR was 96.6% for primary procedures and 96.1% for revision procedures in 2021 [13]. All citizens in Denmark receive a unique 10-digit personal identifier (CPR number), which makes possible exact individual-level record linkage across time and registers [10].

### Study cohort

We defined our population as all knees in Denmark that received a primary knee arthroplasty from January 1, 1998 to December 31, 2021, and by default each person could contribute with 2 knees. All types of knee arthroplasties including TKAs and various types of unicompartmental knee arthroplasties (UKA) were included. We identified all primary knee arthroplasties performed in this period from the DKAR and DNPR. The DKAR and DNPR were queried in the same period for all revision knee arthroplasties performed on the identified primary knees. The data from the different registers was merged. Missing data on the CPR number, laterality of procedure, duplicate procedures, and procedures where the sequence of revision procedures could not be determined (such as date of death occurring before a procedure) was removed. Revisions were identified from the primary cohort and ordered sequentially for each knee.

### Definitions

Revision was defined as any surgical procedure with the exchange, removal, or addition of an arthroplasty component [4]. However, knee arthrodesis and amputation were not included in this study. Revisions were subsequently grouped as being “major revisions” if the tibial and/or the femoral component were exchanged [12,14]. However, insertion of spacer was not regarded as a major revision. In addition to the “major revision,” ”all revisions” included liner exchange, patellar resurfacing, and insertion of spacers.

We used the term “n-grade revision” to specify the number of knee-specific revisions performed, i.e., the second revision for a knee was defined as a 2nd-grade revision and a third revision for 1 knee was defined as a 3rd-grade revision [15].

### Statistics

Patient characteristics for the primary cohort and a flowchart describing data collection were created [16]. We used tables to describe the annual incidence of primary procedures and n-grade revisions over time. Kaplan–Meier plots with 95% confidence intervals (CI) were created to visualize the year-by-year survival likelihood of primary procedures and n-grade revisions over the whole of the observation period. Revision was defined as the event of interest. Observations were censored by the date of death, emigration, and survival to January 1, 2022. In addition, we calculated the absolute incidence of n-grade revisions for primary procedures performed in 2 periods: 1998–2009 and 2010–2021. The survival of primary and n-grade revisions for the 2 periods was calculated and plotted. Only 3 or more observations were shown to ensure patients’ anonymity, as counts below 3 are considered potentially personally identifiable information by Statistics Denmark. CI was calculated for each estimate and depicted in the survival plots. Wald’s test was used to estimate differences between survival probabilities [17]. RStudio version 4.2 was used for all calculations (R Foundation for Statistical Computing, Vienna, Austria).

### Approvals, ethics, data sharing, funding, and disclosures

Approval concerning data storage and analysis was obtained from the Capital Region Head of Knowledge Centre on behalf of the Danish Data Protection Agency (case number: P-2022-711). No approval was required from the regional ethical committee for conducting register studies in Denmark. Access to data is available from Statistics Denmark and the Danish Knee Arthroplasty Register. Access to data is, however, restricted and requires application and approval. The study was funded by Rigshospitalets Forskningspulje, sundhedsdonationer.dk, and Aase and Ejner Danielsens. The Section for Biostatistics and Evidence-Based Research, the Parker Institute, Bispebjerg and Frederiksberg Hospital is supported by a core grant from the Oak Foundation (OCAY-18-774-OFIL). The authors report no conflict of interests. Complete disclosure of interest forms according to ICMJE are available on the article page, doi: 10.2340/17453674.2024.41257

## Results

**Figure 1:**
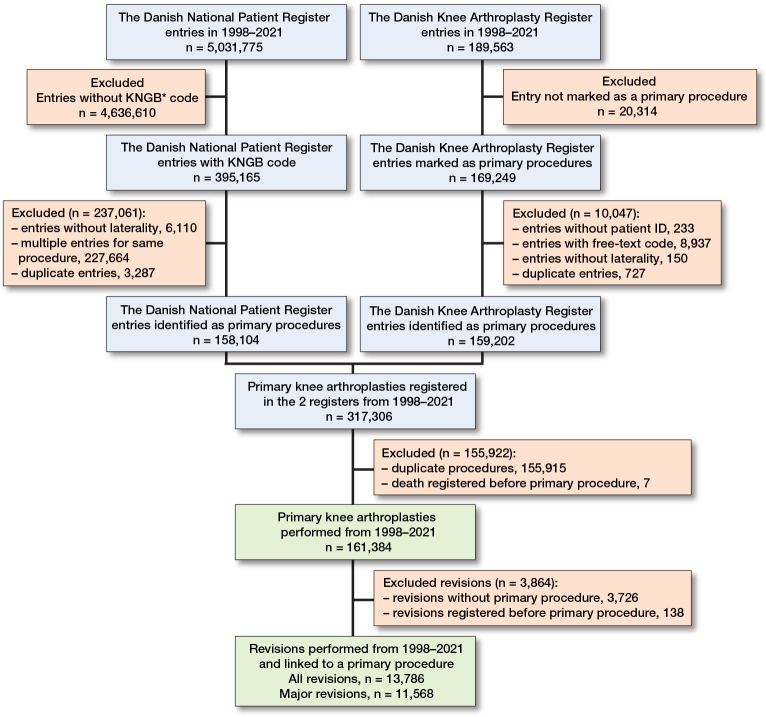
Flowchart describing data-collection process.

5,031,775 entries on all surgical procedures were available from the DNPR and 189,563 entries from the DKAR from January 1, 1998 to December 31, 2021. After the exclusion of irrelevant procedures, entries without personal ID and entries without laterality, there remained a primary knee arthroplasty population of 161,384 knees performed in Denmark between 1998 and 2021 (Figure 1). Of these, 8,655 procedures were not found in the DNPR, and 4,438 procedures were not found in the DKAR. The median age at the time of the primary procedure was 68 years (interquartile range 61–75) and 95,864 (59.4%) of the knees related to women (Table 1). The most common procedure was TKA with 137,254 (85.0%) procedures followed by medial UKA with 16,946 (10.5%) procedures. 135,010 (83.7%) of the procedures were performed in public institutions.

**Table 1:** Demographics for primary knee arthroplasty patients at baseline (i.e., at the time of primary knee arthroplasty). Values are count (%) unless otherwise specified

	Total n = 161,384	1998–2009 n = 55,054	2010–2021 n = 106,330
Age, median (IQR)	68 (61–75)	68 (61–75)	68 (61–75)
Females	95,864 (59)	34,285 (62)	61,579 (60)
TKA	137,254 (85)	50,384 (92)	86,870 (82)
Medial UKA	16,946 (11)	1,827 (3.3)	15,119 (14)
Lateral UKA	871 (0.5)	332 (0.6)	539 (0.5)
Patellofemoral UKA	1,909 (1.2)	287 (0.5)	1,622 (1.5)
Unspecified UKA	1,025 (0.6)	231 (0.4)	794 (0.7)
Unspecified implant	3,379 (2.1)	1,993 (3.6)	1,386 (1.3)
Public institutions	135,010 (84)	45,205 (82)	89,805 (85)
Private institutions	26,324 (16)	9,849 (18)	16,475 (16)
Unknown institution	50 (0.0)	0 (0.0)	50 (0.0)

### All revisions (Figure 2A)

The absolute incidence of all n-grade revisions was: 10,638 (6.6%) 1st revisions, 2,148 (1.3%) 2nd revisions, 624 (0.3%) 3rd revisions, 223 (0.1%) 4th revisions, and 153 (0.1%) revision with a grade > 4. The maximal grade of revision performed during the observation period was 11.

**Figure 2:**
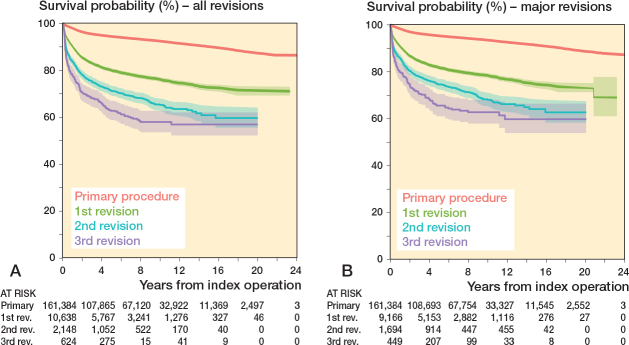
Kaplan–Meier plots of the implant survival of all revisions (A) and major revisions (B). The number at risk at each timepoint is shown below the graph.

The 10-year revision-free survival of 161,384 primary procedures was 92.3% (CI 92.2–92.5) with 49,038 knees remaining at risk after 10 years. 10,638 1st-revision knees had a 10-year revision-free survival of 75.9% (CI 74.9–76.9) with 2,130 knees remaining at risk after 10 years. For the 2nd and 3rd revisions the 10-year revision-free survival was 65.1% (CI 62.6–67.6) and 57.8% (CI 53.4–62.5), respectively. The 10-year revision-free survival probability decreased significantly for each grade of revision except for the 2nd and 3rd revision.

### Major revisions (Figure 2B)

We identified 13,786 revisions of which 11,568 (83.9%) were major revisions. The absolute incidence of the number of major n-grade revisions was: 9,166 1st revisions, 1,694 2nd revisions, 449 3rd revisions, 148 4th revisions, and 111 procedures with a grade > 4.

The 10-year survival without major revision of 161,384 primary procedures was 93.4% (CI 93.2–93.5) with 49,573 knees remaining at risk after 10 years. Knees that had had a major 1st revision had a 10-year survival without major revision in 78.3% (CI 77.3–79.3) with 1,878 cases remaining at risk after 10 years. The 10-year survival without major revision for knees that had had 2 or 3 major revisions were 67.9% (CI 65.2–70.8) and 62.7% (CI 57.8–67.9), respectively. The 10-year implant survival probability without major revision decreased significantly for each grade of revision except for the 2nd and 3rd revision.

### Comparison 1998–2009 vs 2010–2021

**Figure 3:**
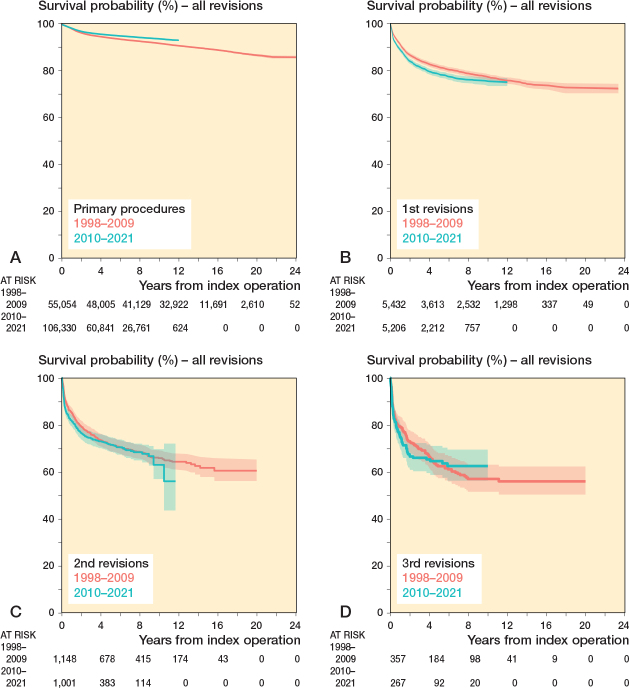
Kaplan–Meier plots of the implant survival of primary procedures (A), 1st revisions (B), 2nd revisions (C), and 3rd revisions (D). Comparison of 2 periods: 1998–2009 and 2010–2021. The number at risk at each timepoint is shown below the graph. The procedures performed 1998–2009 have up to 24 years of follow-up, whereas the procedures performed 2010–2021 have up to 12 years of follow-up.

55,054 primary knee arthroplasties were performed from 1998 to 2009 and 106,330 from 2010 to 2021 (Table 2). The 10-year implant survival probabilities of primary knee arthroplasties were 91.4% (CI 91.1–91.6) in 1998–2009 and 93.3% (CI 93.1–93.5) in 2010–2021, corresponding to a difference between periods of 2.2% (CI 1.9–2.5; P < 0.001) (Figure 3A). The 10-year implant survival probabilities of 1st revisions were 77.2% (CI 76.0–78.4) in 1998–2009 and 75.4% (CI 73.8–76.9) in 2010–2021, corresponding to a difference between periods of –2.4% (CI –4.2 to –0.4; P = 0.02) (Figure 3B). The 10-year implant survival probabilities of 2nd revisions were 65.8% (CI 62.8–68.9) in 1998–2009 and 62.9% (CI 56.9– 69.6) in 2010–2021, corresponding to a difference between periods of 4.4% (CI –2.5 to 10.9; P = 0.2) (Figure 3C). The 9-year implant survival probabilities of 3rd revisions were 57.0% (CI 51.5– 63.0) in 1998–2009 and 62.4% (CI 56.2–69.5) in 2010–2021, corresponding to a difference between periods of 8.8% (CI –0.4 to 15.8; P = 0.06) (Figure 3D).

**Table 2:** Absolute incidence of primary procedures and revisions, 1998–2009 vs 2010–2021. Values in parentheses indicate the percentage revised from the previous grade of revision

	1998–2009	2010–2021
Primary procedures	55,054	106,330
1st revision	2,857 (5.2)	5,206 (4.9)
2nd revision	476 (17)	1,001 (19)
3rd revision	119 (25)	267 (27)
4th revision	26 (22)	88 (33)
> 4 revisions	3 (12)	60 (68)

All procedures, including revisions, are performed in the given period.

## Discussion

This study evaluated the incidence and implant survival of revised knee arthroplasties with a special emphasis on multiply revised knees. 0.3% of the 161,384 primary knee arthroplasties had 3 or more subsequent revisions. The absolute incidence of all n-grade revisions was: 10,638 1st revisions (6.6% of 161,384 primary arthroplasties), 2,148 2nd revisions (1.3%), 624 3rd revisions (0.4%), 223 4th revisions (0.1%), and 153 procedures with a grade of revision above 4 (0.1%). We found that the survival probability was 2.2% higher for primary procedures when performed in 2010–2021, but that the survival probability was 2.4 % higher for 1st revisions when performed in 1998–2010.

The incidence of 1st, 2nd, and 3rd knee arthroplasty revisions has been investigated in the National Joint Registry (NJR) covering most of the United Kingdom from 2003 to 2018 [5,18]. The study identified 1,148,855 primary procedures and 75,881 revisions. However, only 33,292 revisions could be linked to a primary procedure. The study identified 33,292 1st revisions, 3,575 2nd revisions, and 574 3rd revisions. Considering the size of the primary population, this study finds more n-grade revisions performed in a smaller primary population. A possible cause for this difference may be that, in this study, primaries were identified first and revisions identified prospectively. By combining the Danish National Patient Register (DNPR) and the Danish Knee Arthroplasty Register (DKAR), the current study has complete coverage of all arthroplasties conducted in Denmark during the study period. However, our results may also vary due to potential differences of age, sex, and comorbidity distributions between the populations of Denmark and the United Kingdom.

The survival probabilities of n-grade revisions were observed to decrease with each consecutive revision. This finding is an important reminder for surgeons and patients during the shared decision on whether to undergo repeated revisions. The survival of major revisions was notably higher, as anticipated given the exclusion from consideration of minor revisions. The survival likelihood of a major revision is interesting for surgeons, but may not be a relevant outcome for patients, who are more interested in the total number of surgeries performed on their knee. Thus, we chose to analyze all revisions and major revisions to include all returns to the operating theatre and to describe the number of revisions without counting a 2-stage revision as 2 different revisions. Regarding the survival of all revisions, our findings were in accordance with the findings from the NJR of Deere et al. They found that, for each consecutive revision, the time to the next revision decreased by almost 50% [5].

By comparing the early and late study period (1998–2010 vs 2010–2021), we found that the number of procedures performed and the grades of revision increased during the latter period. We found that more revisions were performed in the latter half of the observation period, despite a shorter period of follow-up. One explanation could be an increased proportion of revisions due to infection, such as DAIR procedures. This has been shown in a previous study and could explain why the survival of 1st revisions was lower in the latter half of the observation period [19]. An increased number of DAIR procedures, which might fail in up to 25% of cases, would generate an increased number of multiple revisions [19]. In addition, for this analysis we included insertion of spacers, so changes in the frequency of 1- or 2-stage procedures over the observation period would impact our results. Due to decades of advancement in surgical technique, implant design, and also higher patient demands, it is possible that knee arthroplasty surgeons in the latter half of the observation period were more willing to try a revision procedure in cases where they formerly considered a salvage procedure, such as amputation or arthrodesis. This assumption is supported by a Danish study describing a declining incidence of knee arthrodesis and femur amputation during the observed period [20,21]. However, we have no data on the surgeons’ willingness to perform a revision, so this is merely hypothetical. Yet, these results should be interpreted with caution as the length of follow-up was longer for those patients who received their primary implant in the earlier observation period compared with the patients who received their primary implant in the latter observation period.

### Limitations

First, as a register-based analysis there is an inherent risk of sampling bias. As the number of patients gets smaller as the grade of revision increases, the problems of registry inaccuracies and patient-specific factors become more apparent. One issue was the low completeness rate of revisions in the DKAR at the beginning of the observation period. For this reason, we included data from the DNPR, where data collected during the whole of the observation period was required by law to be reported. Second, the low number of patients undergoing repeated revisions makes the study vulnerable to being under-powered when evaluating long-term survival. Extraordinarily large registers, or pooling of multiple registers, are needed to make robust scientific investigations of patients undergoing several re-revisions. Lastly, patient-, surgeon-, and implant-related factors might have changed over the observation period, and these factors may inhibit comparisons across different national populations. However, as a large national register with a long observation period is the only viable method for making investigations of this patient population this must be accepted. Medical tourism may have had an effect on our results. However, this is believed to be an extremely rare event in relation to revision knee arthroplasty in Denmark due to the well-functioning and publicly funded healthcare system.

### Conclusion

We showed an incidence of 0.3% of primary knee arthroplasties performed in Denmark from January 1, 1998, to December 31, 2021 being revised 3 or more times. The likelihood of 10-year implant survival for any 1st and 3rd revisions was 92.3% (CI 92.2–92.5) and 57.8% (CI 53.4–62.5). In perspective, we found that implant survival decreases for each subsequent revision and, thus, careful consideration should be applied before advising multiply revised patients to undergo yet another revision.

## Notes

JH, MMP, RC, AEG, and AO designed the study. JH completed data collection and data analysis under supervision of AEG. All authors contributed to data interpretation. JH wrote the initial draft and all authors contributed to revision and acceptance of the final manuscript.

The authors would like to thank Sabrina Mai Nielsen for assisting in creating the statistical analysis plan.

Handling co-editors: Marianne Westberg and Philippe Wagner

*Acta* thanks Ove Nord Furnes and Peter Lawrence Lewis for help with peer review of this manuscript.
